# Development of DNA Aptamers to Visualize Release of Mycobacterial Membrane-Derived Extracellular Vesicles in Infected Macrophages

**DOI:** 10.3390/ph15010045

**Published:** 2021-12-29

**Authors:** Soonjyoti Das, Sapna Jain, Mohd Ilyas, Anjali Anand, Saurabh Kumar, Nishant Sharma, Kuljit Singh, Rahul Mahlawat, Tarun Kumar Sharma, Krishnamohan Atmakuri

**Affiliations:** 1 Aptamer Technology and Diagnostics Laboratory (ATDL), Multidisciplinary Clinical and Translational Research Group (MCTR), Translational Health Science and Technology Institute, Faridabad 121001, Haryana, India; soonjyotidas.jyoti9@gmail.com (S.D.); anjalianand@thsti.res.in (A.A.); nishant@thsti.res.in (N.S.); kuljitniper@gmail.com (K.S.); rahulmahlawat40@gmail.com (R.M.); 2 Bacterial Pathogenesis Laboratory, Infection and Immunology Group, Translational Health Science and Technology Institute, Faridabad 121001, Haryana, India; sapna.jain@thsti.res.in (S.J.); m.ilyas@thsti.res.in (M.I.); saurabhkandwal@hotmail.com (S.K.); 3 Special Centre for Molecular Medicine, Jawaharlal Nehru University, New Delhi 110067, Delhi, India; 4 Department of Biotechnology, Jamia Hamdard, New Delhi 110062, Delhi, India; 5 Clinical Microbiology Division, CSIR-Indian Institute of Integrative Medicine, Jammu 18001, Jammu and Kashmir, India

**Keywords:** mycobacteria, extracellular vesicles, aptamers, nucleic acids, SELEX, ALISA, tuberculosis

## Abstract

Extracellular vesicles (EVs) have emerged into a novel vaccine platform, a biomarker and a nano-carrier for approved drugs. Their accurate detection and visualization are central to their utility in varied biomedical fields. Owing to the limitations of fluorescent dyes and antibodies, here, we describe DNA aptamer as a promising tool for visualizing mycobacterial EVs in vitro. Employing SELEX from a large DNA aptamer library, we identified a best-performing aptamer that is highly specific and binds at nanomolar affinity to EVs derived from three diverse mycobacterial strains (pathogenic, attenuated and avirulent). Confocal microscopy revealed that this aptamer was not only bound to in vitro-enriched mycobacterial EVs but also detected EVs that were internalized by THP-1 macrophages and released by infecting mycobacteria. To the best of our knowledge, this is the first study that detects EVs released by mycobacteria during infection in host macrophages. Within 4 h, most released mycobacterial EVs spread to other parts of the host cell. We predict that this tool will soon hold huge potential in not only delineating mycobacterial EVs-driven pathogenic functions but also in harboring immense propensity to act as a non-invasive diagnostic tool against tuberculosis in general, and extra-pulmonary tuberculosis in particular.

## 1. Introduction

The generation of extracellular vesicles (EVs) is widely reported across prokaryotes, eukaryotes and archaea [1,2]. Typically, bacterial-generated EVs are spherical, ~10–350 nm in diameter and contain a subset of their cellular contents [1,2,3,4]. Once considered as mere cellular artefacts or trash bags that aid in the disposing of misfolded, degraded and unwanted molecules [2,3,4,5], EVs are now reported to facilitate several intercellular communications, disease physiology, pathogenesis and antimicrobial resistance by transporting a wide range of bioactive molecules including enzymes, DNA, RNA, proteins and toxins [1,2,3,4,5,6,7,8,9,10,11,12,13,14,15,16,17]. The EVs of several pathogens, including *Salmonella typhi*, *Mycobacterium tuberculosis* and *Neisseria meningitidis*, trigger pro-inflammatory cytokines, indicating their inherent immunomodulatory properties [18,19,20]. Consequently, bacterial EVs are now either utilized as novel vaccines [21] or evaluated as potential vaccine, diagnostic and therapeutic candidates [17].

Until early 2000, most bacteriologists presumed that EVs were a characteristic feature of Gram-negative (G-ve) bacteria and thus required an outer membrane for generation and release. Consequently, any observations of EVs released by mycobacteria were considered erroneous and largely attributed to contaminating axenic cultures with fast-growing G-ve bacteria, despite no detection of the proposed contaminants. However, between 2007 and 2015, different groups reported that mycobacteria, too, generate EVs [22,23,24,25], and EVs from pathogenic *Mycobacterium tuberculosis* (Mtb) have the potency to act as a vaccine candidate [23,24].

First reported in 2007, mycobacterial EVs (mEVs) are now considered to be naturally secreted by both pathogenic and non-pathogenic mycobacteria under all growth conditions tested and predicted to do so during infection [22,23,24]. Their proteomic and lipidomic analyses indicate the presence of glycolipids, lipoproteins, surface, periplasmic and cytosolic proteins and enzymes. [23,24,25]. When ingested by macrophages, in a TLR-2 dependent fashion, surface and lipoproteins in the mycolic acid layer of mycobacterial EVs trigger the generation of pro-inflammatory cytokines. Since the induced cytokines profile is similar to those observed by several potential TB vaccine candidates and BCG, mEVs have been explored in mice as a potential alternate and/or as subunit vaccine candidates [23,24]. Despite several roles in TB pathogenesis that are speculatively attributed to mEVs, most mycobacteriologists to this day still consider mEVs as mere in vitro broth culture artefacts and hence their natural release in macrophages remains controversial.

To evaluate this, we set out and developed an aptamer tool that specifically recognizes mycobacterial-derived EVs that we enriched from large volumes of axenic, broth mycobacterial cultures. Here, though we primarily utilize it for visualizing both the internalized mEVs and those released by infecting mycobacteria, we predict that this tool has huge potential in not only delineating the role of mEVs in vitro and ex vivo but also in acting as a potential diagnostic tool against mycobacterial EVs in tuberculosis (TB) patients.

Given that EVs are very small (~10–350 nm in diameter), their best visualization is possible through electron microscopy [1,2,3,4,17,22]. However, because of the complexity involved in the preparation of samples for viewing under electron microscopy, limited access to such expensive technology and limitations to study EVs in action in real-time with most fluorescent microscopes, several groups have employed alternate means to visualize EVs. Broadly, groups have mostly employed either fluorescent dyes or antibodies [12,13,14,15,16,17,26,27,28,29]. Fluorescent dyes are usually non-specific or cross-reactive, while antibodies often suffer from reproducibility issues. Interestingly, in a validation survey of 2008 conducted by Human Protein Atlas [30], of >5000 antibodies (procured from 51 different commercial sources), ~51% failed to recognize their targets in different assays. More recently, from 16 antibodies (from seven vendors) against C9ORF72—a human protein specific to amyotrophic lateral sclerosis (ALS)—only one worked well in immunofluorescence, while two worked for Western blotting [31]. These reports imply that one needs to purchase different antibody sources for different assays. Moreover, polyclonal antibodies are infamous for evincing high batch-to-batch variation, as only 0.5–5% of total sera antibodies are often specific to an intended target [32]. Further, despite the affinity purification of antibodies, due to the inefficiency in eliminating most cross-reactive species, the “purified” lot often exhibit varied sensitivity and specificity [33].

To address these and other limitations of antibodies [32,33], in recent years, nucleic acid aptamers have emerged as a superior alternate. Aptamers are synthetic nucleic acid molecules that not only recognize and bind their target “epitopes” (sites) with high sensitivity and specificity but also rarely evince batch-to-batch variation [34,35,36,37,38]. Moreover, as aptamers are amenable and tolerate various modifications, a single aptamer can be used for a variety of applications, including, but not limited to, Aptamer Linked Immobilized Sorbent Assay (ALISA), Western blotting and microscopic examination. Considering these advantages in the current work by utilizing the in vitro evolution strategy viz. Systematic Evolution of Ligands through Exponential enrichment (SELEX), we identified mycobacterial-derived EVs (mEVs)-specific aptamers and evaluated them for their binding affinity and specificity. We then exploited the ability of the best performing aptamer candidate to detect mEVs in THP-1 macrophages post-mycobacterial infection. Our detailed microscopic analyses indicate that the chosen aptamer candidate (aptamer-21, later referred to as apt-21) is an excellent tool for not only visualizing mEVs that the macrophages take up upon their exposure to mEVs, but also for detecting them after their release by the infecting mycobacteria within macrophages. Our work, for the first time, visually demonstrates that *Mycobacterium tuberculosis* (Mtb) can efficiently release EVs upon infecting macrophages and that the released mEVs quickly spread to other cellular regions away from the site of bacterial localization.

## 2. Results

Until the report by Marsollier et al. in 2007 [22], no one had predicted that mycobacteria are capable of generating EVs. However, in the last 12 years, several groups have reported that, similar to other bacteria, mycobacteria, too, generate and release EVs [2,23,24,25]. Despite such reporting, surprisingly, to this day, most mycobacteriologists continue to consider mEVs as mere artefacts of in vitro-grown axenic broth and plate cultures.

To demonstrate that mycobacteria can generate and release EVs even after infecting macrophages, we initially set out to first enrich Mtb_Rv_ EVs in vitro and then use the necessary quantity to generate a high-titer polyclonal antibody against them. However, we soon realized that this required substantial culturing effort (~60–80 L) as routine Mtb_Rv_ mEVs enrichment only yields 80–120 µg protein equivalent per 2 L of axenic culture. Further, generating EVs from pathogenic Mtb_Rv_ required a lot more culturing time (approx. five to six weeks on roller bottles) and continuous access (of several hours) to centrifuges in a BSL3 facility, which was often challenging.

Thus, to bypass these and other experimental and practical handling hurdles, we explored the aptamer approach. We screened for high-affinity binding ssDNA aptamers that only required ~100 µg protein equivalent of mEVs for both screening the aptamer library and, thereafter, shortlisted them. Towards this, we utilized the enriched EVs of *M. smegmatis* (Msm) because: (i) ~50% of Mtb (H37Rv)-derived EVs proteome overlapped with that of Msm EVs (an independent manuscript submitted for peer review to a different journal); and (ii) of ease in enriching them in a BSL2+ setting.

### 2.1. Screening DNA Aptamer Library by SELEX Aids in Selection of High-Affinity Binders That Specifically Target mEVs

**Figure 1:**
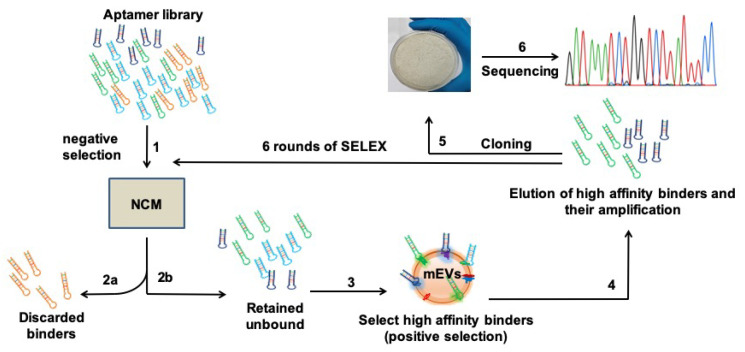
Schematic representation of Systematic Evolution of Ligands by EXponential enrichment (SELEX) process employed for shortlisting high-affinity aptamers specific to mycobacterial EVs. Aptamer library was first (step 1) negatively selected on a blank nitrocellulose membrane (NCM). The bound aptamers (step 2a) were discarded, and unbound aptamers (step 2b) were taken forward to screen for high-affinity binders to *Mycobacterium smegmatis* (Msm) EVs (step 3). The bound high-affinity binders were then eluted and amplified (step 4). Steps 1 to 4 were followed for an additional six rounds of SELEX to shortlist the best binders to mEVs. Finally, the shortlisted binders were cloned (step 5) and sequenced (step 6).

By employing SELEX, a Systematic Evolution of Ligands by EXponential enrichment approach, we first screened our well-optimized DNA aptamer library [37] against the EVs of Msm. Using a fresh, plain and uncoated nitrocellulose membrane (NCM, Step 1, Figure 1), we first performed a subtractive SELEX strategy and screened out the non-specific NCM-binding aptamers (Step 2a, Figure 1). Collecting the unbound (Step 2b, Figure 1), we then screened for DNA aptamer molecules that would specifically bind to Msm-derived EVs. The Msm-derived EVs were enriched (as reported by others [23,24]) and characterized by Transmission Electron Microscopy (TEM; Figure S1) and Nanoparticle Tracking Analysis (NTA; Figure S2). We pre-coated the EVs onto freshly equilibrated NCM (Steps 3 and 4, Figure 1), eluted the mEVs-bound aptamers and PCR-amplified them (Step 4, Figure 1, detailed protocols in Materials and Methods).

We then reiteratively screened the amplified pool of mEVs-specific binders for six additional rounds (steps 1–4) with high binding stringency and achieved saturated binding of the efficient binders right from the first round to the last (Figure S1A), thus shortlisting 15 mEVs-specific high-affinity binders (Figure S1B). These strongly bound binders were individually eluted, cloned into a TA cloning vector (step 5, Figure 1) and sequenced (step 6, Figure 1). The sequenced aptamers were subjected to CLUSTAL W analysis. As evident from Figure S1B, all shortlisted mEVs-binding aptamers were primarily clustered into two preponderant groups. Interestingly, the majority (~94%) of them clustered together in Group-1. Group-1 constituted two sub-groups viz. SG-1 and SG-2, with SG-2 being predominant (78%, Figure S1B).

**Figure 2:**
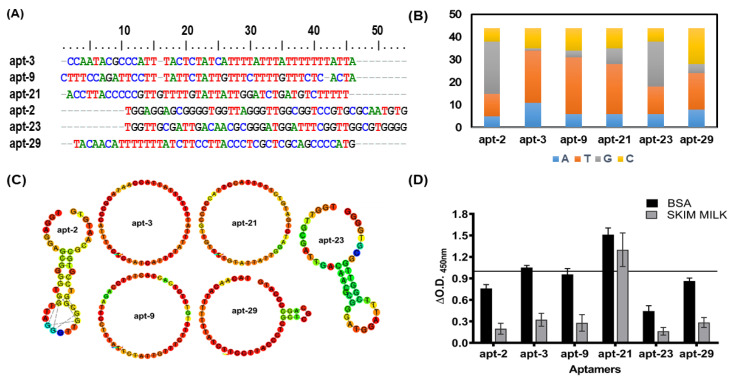
Evaluation of shortlisted mEVs-specific high-affinity binders: (**A**) CLUSTAL W generated primary sequence alignment of high-affinity binders as listed to the left (black); (**B**) differential distribution of nucleotides (numbers, *Y*-axis) across each of the shortlisted high-affinity binders (X-asis); (**C**) secondary structure (RNA fold using DNA parameters, generated using a web-based tool [39]) of SELEX derived high-affinity binders; (**D**) equal numbers of Msm-specific EVs were used to determine the binding affinity (A_450nm_, *Y*-axis) to different aptamers (*X*-axis) under two blocking conditions viz. 5% BSA (black bars) and 5% skim milk (grey bars). Variance is represented as mean ± SD.

Based on the primary nucleotides sequence homology data, we selected six representative aptamer candidates (apt-2, -3, -9, -21, -23 and -29) for further characterization (Figure 2A). Interestingly, these ssDNA molecules not only showed varied distribution of different nucleotides (Figure 2B) but also variable two-dimensional structures (Figure 2C). While apt-3, -9 and -21 displayed only loop-like structures, apt-29 exhibited a large and a small (3-nucleotide) loop (Figure 2C). Similarly, apt-2 and -23 exhibited relatively complex secondary structures containing a long stem in addition to loops (Figure 2C).

### 2.2. Aptamer-Linked Immobilized Sorbent Assay Helps Screen Short-Listed Aptamers for Best High-Affinity Binders

Employing ALISA (aptamer-linked immobilized sorbent assay), we evaluated the shortlisted six representative aptamer candidates (apt-2, -3, -9, -21, -23 and -29; Figure 2A) for their binding propensity to mEVs (Figure 2D). Since blocking conditions do influence the binding propensities, we assessed their binding ability to mEVs under two blocking conditions viz. 5% bovine serum albumin (BSA) and 5% skim milk. Though all shortlisted aptamers evinced good binding to Msm-specific mEVs, the binding intensity was relatively higher when BSA was used as the blocking reagent (Figure 2D). Therefore, for all our subsequent analyses with these aptamers, we used BSA as the blocking agent. Among the six efficient binders, apt-3 and -21 exhibited the highest (O.D. A_450nm_ of >1) binding intensities and hence were used for all downstream characterizations and analyses. Interestingly, apt-21 binding intensity remained unaltered regardless of the blocking agent used (Figure 2D).

### 2.3. Aptamer-3 and -21 Exhibit High Specificity to Mycobacterial EVs

**Figure 3:**
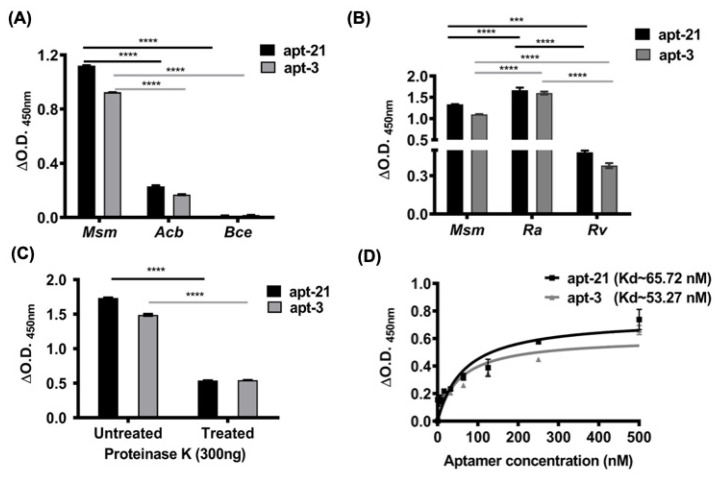
Aptamers-3 and -21 are specific to mycobacterial EVs and bind to surface proteins with very high affinity. (**A**) Equal protein equivalent of EVs from mycobacteria (*M. smegmatis*; Msm), Gram-negative (*A. baumannii*) and Gram-positive (*B. cereus*) bacteria (*X*-axis) were screened for binding intensities (A_450nm_; *Y*-axis) to apt-3 (grey bars) and -21 (black bars). (**B**) Equal protein equivalent of EVs from non-pathogenic (Msm), attenuated (Ra), and virulent (Rv) (both Mtb) mycobacteria (*X*-axis) were screened for binding intensities (A_450nm_; *Y*-axis) to apt-3 and -21. (**C**) Equal protein equivalent of Msm-derived EVs treated or untreated with proteinase K (*X*-axis) were assessed for impact of proteinase K treatment on aptamer binding (A_450nm_; *Y*-axis) to apt-3 and -21. (**D**) Increasing concentrations (2–500 nM; *X*-axis) of apt-3 and -21 were bound to equal protein equivalent of Msm-specific EVs and screened for binding affinity (A_450nm_; *Y*-axis) to determine dissociation constant (Kd). Comparative statistical analyses were performed using ANOVA. *** *p* < 0.0006, **** *p* < 0.0001. ANOVA was performed on values obtained from replicates used in two independent experiments.

Employing ALISA, we next assessed if apt-3 and -21 exhibited high specificity to only mEVs. In addition to Msm-derived EVs, we also enriched EVs of: (a) Mtb_Rv_, pathogenic and Mtb_Ra_, attenuated; (b) *Acinetobacter baumannii* (Acb), a representative Gram-negative bacterial pathogen; and (c) *Bacillus cereus* (Bce), a representative Gram-positive bacterial pathogen [40,41]. Coating equal protein equivalents of EVs on 96 well plates, we first assessed the binding specificity of apt-3 and -21 to EVs derived from G-ve Acb and Bce bacteria and compared that to their binding specificity to EVs of Msm (Figure 3A). As predicted, both apt-3 and -21 did not exhibit any significant binding to EVs derived from *Acinetobacter baumannii* and *Bacillus cereus* (Figure 3A). In contrast, as expected, both apt-3 and -21 were not only bound with high intensity to Msm-derived EVs (Figure 3A,B) but also bound significantly to Mtb-derived EVs (Figure 3B). Affinity to EVs derived from Mtb_Ra_ (attenuated) was higher as compared to EVs derived from Mtb_Rv_ (pathogenic) (Figure 3B). These findings demonstrate the high specificity of aptamer-3 and -21 towards only mycobacterial EVs.

### 2.4. Significant Binding by Aptamers-3 and -21 to mEVs Is through Surface Proteins

Several groups have suggested that the surface of mEVs not only contains a phospholipid mono or bilayer, but also an outer mycolic acid layer together with its associating and traversing surface proteins [23,42,43]. Consequently, we predicted that the aptamers we shortlisted might bind to surface proteins, lipoproteins, glycoproteins and/or lipids. To determine if apt-3 and -21 bound largely to surface-exposed proteins of mEVs, we treated intact Msm-derived EVs to mild Proteinase K (300 ng) and compared their binding intensities with untreated EVs. Interestingly, both apt-3 and -21 exhibited significantly reduced binding (~70%) to Proteinase K treated mEVs (Figure 3C), suggesting that the primary targets for the aptamers binding are surface proteins of Msm-derived EVs.

### 2.5. Aptamer-3 and -21 Exhibit Nanomolar Levels of Dissociating Constant and Thus Very Tight Binding

The dissociation constant (Kd) determines the affinity with which aptamer binds to its target sites. Thus, to determine the strength of binding of the apt-3 and -21 to their target sites on the surface of mEVs of Msm by employing ALISA and using increasing concentrations of aptamers and constant protein equivalent of mEVs, we estimated their Kd values (Figure 3D). As expected, the Kd values of both apt-3 and -21 were in the low nanomolar range (53–65 nM), indicating very tight binding affinity to mEVs (Figure 3D).

### 2.6. Aptamer-3 but Not -21 Binds Non-Specifically to THP-1 Macrophages

**Figure 4:**
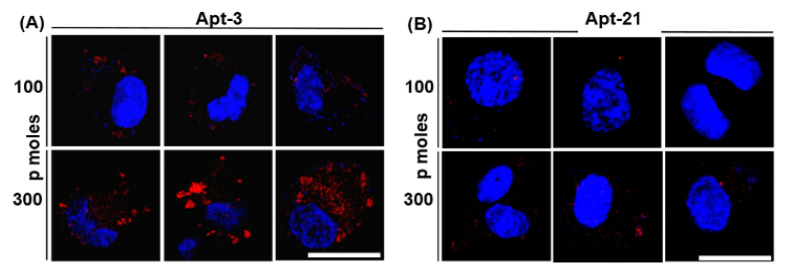
Aptamer 21 *per se* does not bind to THP-1 macrophages. In 2-well chamber slides, Cy5-labelled apt-3 (**A**) and -21 (**B**) were incubated at 100 (**top panels**) and 300 picomoles (**lower panels**) for 4 h with 10^5^ PMA-differentiated THP-1 macrophages at 37 °C and 5% CO_2_. The unbound aptamers were washed off with pre-warmed 1× PBS (pH 7.4). Cells were fixed with BD cytofix/cytopermeabilization kit and mounted with Prolong Gold antifade DAPI to observe under the confocal microscope. Blue, DAPI (nuclear staining of THP-1); Red, apt-3/-21. The images were captured at 60× magnification. White bars in (**A**,**B**) bottom right-most panels indicate scale bar of 20 µm (applies to all 12 panels).

Our primary objective was to utilize the best performing aptamer and visually determine if Mtb is capable of generating and releasing EVs during infection of macrophages. Given the very high affinity and low dissociation constant for both apt-3 and -21, we evaluated which among the two best labelled the released EVs during infection. Towards that, we first evaluated which among the two did not bind to THP-1 per se. We incubated the Cy5-labelled apt-3 and -21 at two different concentrations (100 and 300 picomoles) along with THP-1 macrophages and tested for non-specific binding using fluorescent confocal microscopy. Interestingly, despite exhibiting the lowest dissociation constant and the highest affinity to mEVs, apt-3 bound non-specifically to THP-1 macrophages (Figure 4A). In contrast, at 100 picomoles, apt-21 barely bound to THP-1 macrophages (Figure 4B). Similarly, at 300 picomoles, though it bound a bit non-specifically to THP-1 cells, apt-3 bound to THP-1 with very high non-specificity (Figure 4A,B). Consequently, for all further downstream experiments with THP-1 macrophages, we used only apt-21.

### 2.7. Aptamer-21 Detects mEVs Post Their Uptake by THP-1 Macrophages

**Figure 5:**
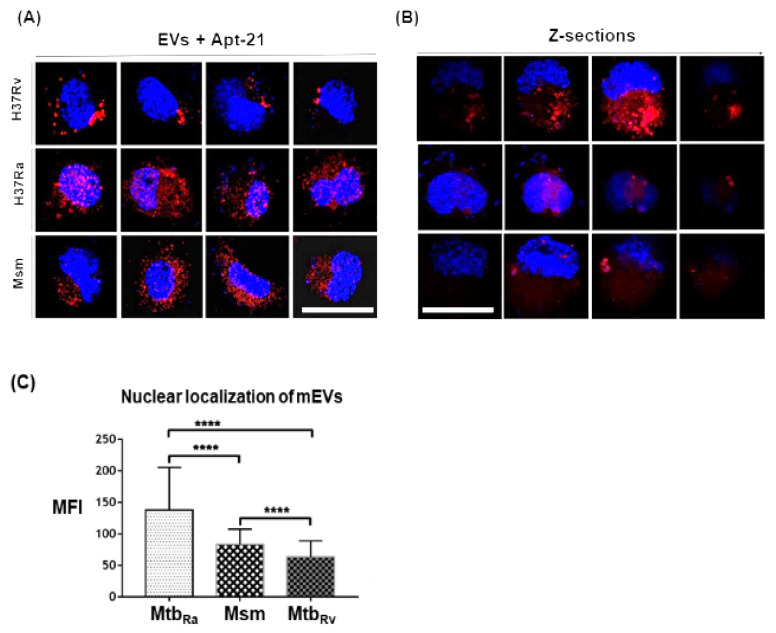
Aptamer-21 detects mycobacterial EVs post uptake by THP-1. (**A**,**B**): PMA-differentiated THP-1 were first incubated with mEVs from three mycobacterial strains (Msm, H37Ra and H37Rv) for 2 h at 37 °C, followed by 2 h exposure with Cy5-labelled apt-21 (100 pm). The images were acquired at 60× using FV 3000 software. (**B**) Z-sections were acquired to assess the localisation of mEVs in the nucleus of THP-1 macrophages. (**C**) mEVs localized to the nucleus were quantified by cellSens Dimension software and a student *t*-test (unpaired) was performed. Mtb_Rv_, H37Rv (pathogenic); Mtb_Ra_, H37Ra (attenuated); Msm, mc^2^155 (*M. smegmatis*, avirulent); MFI, mean fluorescence intensity. **** *p* < 0.0001. The images were captured at 60× magnification. White bars in (**A**,**B**) bottom right and left-most panels indicate scale bar of 20 µm (applies to all 24 panels).

We next evaluated if Cy5-labelled apt-21 could bind specifically to mEVs upon their uptake by THP-1 macrophages. Since the aptamer bound with high affinity to not only the EVs of Msm but also to EVs derived from Mtb strains, Rv (pathogenic) and Ra (attenuated), we tested its ability to detect all three EVs post their uptake by THP-1 macrophages. As expected, we could easily detect the EVs of Msm, Mtb_Ra_ (H37Ra) and Mtb_Rv_ (H37Rv) in THP-1 macrophages post their uptake (Figure 5A). Surprisingly, we also observed a proportion of EVs of Msm, Mtb_Ra_ and Mtb_Rv_ localizing to the nucleus (Figure 5A–C). Of the three strains, the EVs of Mtb_Ra_ localized most to the nucleus, while the EVs of Mtb_Rv_ localized the least (Figure 5A,C). To confirm that they truly had localized to the nucleus, we also captured images in Z-sections. The captured Z-sections demonstrated mEVs localization into the nucleus (Figure 5B).

### 2.8. Aptamer-21 Detect mEVs Released by Mtb Post Infection of THP-1 Macrophages

**Figure 6:**
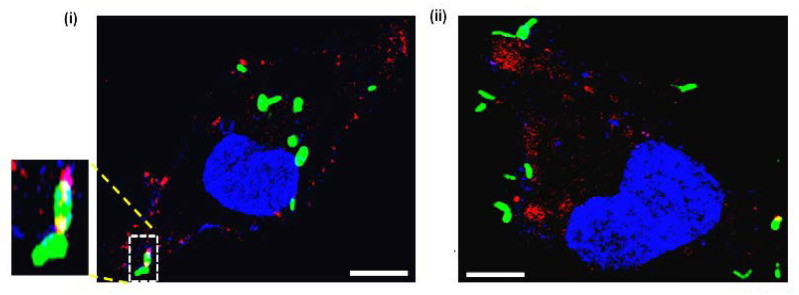
Aptamer 21 detects EVs released by Mtb post-infection in THP-1. PMA-differentiated THP-1 were infected with GFP-expressing Mtb (H37Ra) for 4 h (**i**,**ii** panels). Extracellular Mtb were killed with Amikacin (200 µg/mL), and then the infected THP-1 was incubated for 2 h with Cy5-labelled apt-21 (100 pmoles). Excess aptamer was removed with three washes of pre-warmed 1× PBS (pH 7.4). Cells were fixed with BD cytofix/cyto-permeabilization kit and mounted with Prolong Gold antifade DAPI to observe under the confocal microscope. The images were captured at 60× magnification and zoomed to 120×. Blue, DAPI (nuclear staining); Green, Mtb (H37Ra); Red, apt-21. Inset indicates an enlarged view of bacterium releasing EVs from its surface. White bars in (**i**) and (**ii**) indicate a scale bar of 10 µm. The single-channel images are as in Figure S3.

Given that apt-21 bound specifically to mEVs and detected EVs post uptake by THP-1 macrophages, we assessed whether it would also detect the freshly released mEVs post-infection of THP-1 by Mtb. THP-1 were infected for 4 h with GFP-expressing Mtb_Ra_ and later incubated with Cy5-labelled apt-21 for detecting released EVs of Mtb_Ra_. As expected, Cy5-labelled apt-21 specifically detected mEVs (Figure 6). Interestingly, even within a period of 4 h, most EVs detected were well away from the bacterial surface indicating their release by bacteria. The released EVs were also predominantly distributed across the cytosol of THP-1 (Figure 6; both (i) and (ii)).

## 3. Discussion

To the best of our knowledge, this is the first report on the use of aptamers as a tool to detect and visualize mycobacterial EVs. We initially considered generating and purifying high titer rabbit-derived polyclonal antibodies that were highly specific to EVs derived from Mtb. However, since this strategy required (i) a high quantity of antigen (Msm-derived EVs: ~4 mg) and (ii) large volumes of Mtb_Rv_ broth cultures (~60–80 L) in BSL3 settings, we explored the aptamer-based approach and succeeded in quickly shortlisting a mEVs-specific aptamer (apt-21) with just a fraction (<100 µg) of the antigen amount. Additionally, for shortlisting, instead of using Mtb_Rv_ (pathogenic)-derived EVs as the antigen source, we employed EVs of Msm (avirulent mycobacterium). We did so, as our previous work on the comparative proteome profiling of EVs from different mycobacteria showed a significant overlap of Msm EVs proteome content with both Mtb_Rv_ (pathogenic) and Mtb_Ra_ (attenuated) EVs proteins (~44–50%; *unpublished data—a separate manuscript with the unpublished data is submitted elsewhere for peer review*).

As expected, not only did apt-21 bind efficiently and specifically to Msm-derived EVs (Figure 3A,B), it also exhibited comparable affinity to both Mtb_Rv_ (pathogenic)- and Mtb_Ra_ (attenuated)-derived EVs (Figure 3B), suggesting that it probably recognized overlapping surface protein(s) epitopes across these mycobacteria. The loss of two-thirds of the binding intensity of apt-21 to proteinase K-treated Msm-derived EVs (Figure 3C) indicated that the surface proteins of mEVs were the main target for apt-21. Though apt-3, too, bound in vitro to enriched EVs of Msm with equal affinity (Figure 3D), we abandoned employing it for any of our in vitro infection experiments with THP-1 macrophages (Figure 5 and Figure 6) because of its non-specific binding to macrophage (THP-1) components (Figure 4). Importantly, apt-21 was not only capable of detecting mEVs internalized by THP-1 macrophages (Figure 5A,B), it also detected with ease mEVs released by infecting Mtb_Ra_ into THP-1 macrophages (Figure 6), suggesting that the specificity of the apt-21 was for both in vitro enriched (from broth cultures) and released mEVs (during in vitro infections). The higher binding intensity of apt-21 to Mtb_Ra_-derived over Mtb_Rv_-derived EVs suggested that the potential epitope/aptatope of apt-21 may have been overrepresented on Mtb_Ra_-derived EVs surface. Interestingly, in our comparative analyses of EVs proteome, we consistently found more proteins in Mtb_Ra_ (~1200) as compared to Mtb_Rv_ (~640; manuscript submitted elsewhere, under peer review) suggesting that perhaps the common epitope/aptatope to which apt-21 bound may have been slightly under-represented in the pathogenic strain of Mtb.

Typically, most labs, including ours, routinely grow axenic mycobacterial broth cultures to the mid-logarithmic stage, spin bacteria down and filter off remaining bacteria from the spent media to obtain a culture filtrate that is enriched with the released mEVs, which then can be visualized under scanning and transmission electron microscopes. However, except for the study by Pardos et al. in 2011 [23] discussing the electron micrograph of mycobacteria possibly releasing one or two EVs in vitro during infection, there are no other reports that clearly demonstrate the release of large numbers of EVs by infecting mycobacteria. Consequently, for a long time, EVs have been considered as artefacts of in vitro grown broth cultures and conceived to not be generated and released during infections [44]. In this context, Figure 6 not only demonstrates that EVs are released by infecting mycobacteria (Mtb_Ra_), but also that they spread across the THP-1 macrophages cellular environment away from the site at which the bacteria are located.

Owing to their high affinity, selectivity, negligible batch-to-batch variability, non-toxic nature and amenability to various modifications, nucleic acid aptamers (ssDNA or RNA aptamers) have emerged as a strong chemical rival to antibodies [9,45,46]. A huge body of literature clearly indicates broad applications of aptamers ranging from imaging to clinical diagnostics, cytokine detection, detection of small molecules and much more [5,7,47,48]. Moreover, in recent years utility of aptamers has also been demonstrated to detect EVs from cancer cells by capturing the cells through aptamers targeting CD63, EpCAM, PDGF-β receptor or nucleolin [49,50,51].

The routine way of visualizing EVs is to target their specific antigen(s) using fluorescently labelled antibodies and then observe them under a confocal microscope [20]. However, this process is challenging, especially when the EVs are inside other cellular environments and thus, due to the large size of antibodies, cell permeabilization becomes necessary for their entry into the cell. In contrast to antibodies, aptamers are ~10–100 times smaller [52,53,54] and, owing to such small size, DNA aptamers are considered nanomaterials [55,56]. Their small size facilitates the entry of aptamers into eukaryotic cells without any aided permeabilization. Recent studies clearly indicate that aptamers can easily cross the cell membrane by a variety of mechanisms including, but not limited to, clathrin- and caveolae-mediated endocytosis, macropinocytosis and phagocytosis [57,58]. In this study, we exploited this property of aptamer and demonstrated its utility to visualize mEVs in THP-1 macrophages. Recently, aptamers have been utilized for the detection of outer membrane vesicles of *Escherichia coli* [59], but to the best of our knowledge, this is the first report on the development and application of DNA aptamer for the intracellular visualization of mEVs, especially after their release by the infecting mycobacteria.

Given that we showed (in vitro) that infecting mycobacteria continued to generate and release EVs during infection, we speculate that this may be also true in TB patients. Currently, EVs released by other bacterial pathogens are being explored as an attractive platform for the development of natural theranostics [60,61]. We speculate that our mEVs-specific aptamer may not only help reveal possible EVs functions in vitro and in vivo (animal models) but also act as a potential non-invasive tool for the superior diagnosis of extra-pulmonary TB cases. Quick and accurate diagnosis of extra-pulmonary TB has remained a challenge for a long time [62,63]. Interestingly, in both pulmonary and extra-pulmonary TB patients, urine has been shown to contain EVs with Mtb components [64]. In the near future, we intend to explore if apt-21 may aid in detecting mEVs in the urine of pulmonary and extra-pulmonary TB patients as a novel invasive diagnostic tool.

All pathogenic bacteria thus far evaluated for the generation of outer membrane-derived EVs (OMVs) definitively released them at least when growing in vitro in pure axenic cultures [1,2,3,4,5,6,7,8,9,10,11,12,13,14,15,16,17]. Given the vast literature and emerging diverse role of EVs, an EVs-specific tool such as ours, when conjugated with a fluorescent tag, holds great potential in delineating pathogenesis (in vitro real-time monitoring/visualization, in vivo imaging, identification of aptatope(s); spatio–temporal localization, etc.) in ways not exploited thus far. It can also be exploited in diagnostic labs as a signature tool for fluorescent- and/or biochemical-based detection of pathogen EVs, especially in cases where bacterial detection per se is false well below the limits of detection. Our conservative estimations (by NTA) of bacterial EVs numbers (that we used here) indicate at least 100 to 1000 EVs per bacterium. Despite these estimates coming from axenic cultures, we predict that, especially in clinical settings, this tool provides improved opportunities for the superior detection of pathogenic bacteria via EVs. Though nucleic acid-based detection remains superior in terms of amplification of an otherwise undetectable signal, given the properties of EVs to be transported to parts of the body that have never seen the pathogen directly, EVs detection may provide a distinct advantage. Thus, we predict that aptamer-21 will hold a huge promise in the superior diagnosis of pulmonary and extra-pulmonary TB, perhaps by the use of urine and/or saliva. In theory, this simple diagnostic approach is easily extendable to the detection of almost every infectious disease wherein the causative agent is a bacterium. We can’t help but wonder if the short-listed apt-21 would also detect pathogen signatures in exosomes, i.e., EVs generated by the host in response to pathogen infection. If so, we speculate that this tool will help us estimate the ratio of exosomes and mEVs with Mtb-specific signatures. We are very excited about the potency the emerging world of EVs and aptamers holds in human health, diagnosis and treatment.

## 4. Materials and Methods

### 4.1. Reagents

Optiprep density gradient medium, Proteinase K and Phorbol-12-myristate-13-acetate were purchased from Sigma Aldrich (Burlington, MA, USA). THP-1 cell line was procured from NCCS, Pune, India. Pierce BCA Protein Assay Kit, RPMI 1640 media, Fetal Bovine serum and Penicillin–streptomycin, Nunc™ Lab-Tek™ II Chamber Slide™, Lysotracker Green and Prolong Gold Antifade DAPI Mountant, BD cytofix/cytoperm Fixation/Permeabilisation Kit, MaxiSorp Nunc 96 well plates were all from Thermo Fisher Scientific (Waltham, MA, USA).

All oligonucleotides used in the current study were purchased from Integrated DNA Technology (IDT, Coralville, IA, USA). PCR master mix was procured from Takara, Japan. Nuclease free water was obtained from HI Media Laboratories, India. For all buffers/solutions preparation, milliQ water was used. The aptamers population was cloned in a TA cloning vector (pTZ57R/T vector) from Thermo Fisher Scientific, USA, using InsTA Clone PCR cloning Kit. 7H9, 7H11 and OADC were purchased from BD (Franklin Lakes, NJ, USA) and Luria Bertiani media was purchased from HI Media Laboratories, Mumbai, India. Most chemical reagents were purchased from either Sigma Aldrich, USA or HiMedia Laboratories Pvt. Ltd., Mumbai, India.

### 4.2. Bacterial Strains and Growth Conditions

*Mycobacterium smegmatis* (Msm; avirulent) mc^2^155 and *M. tuberculosis* (Mtb) strains viz. H37Rv (Rv; pathogenic) and H37Ra (Ra; attenuated) were cultured as described in [65]. To enrich EVs of Msm, Rv and Ra, all three mycobacterial strains were initially grown to 1.0 O.D. (at A_600nm_) in 7H9 broth then washed twice at ~3200× *g* for 15 min at RT with equal volumes of freshly prepared Sauton’s media and then sub-cultured at 0.05 O.D. (at A_600nm_) to the required volume in fresh Sauton’s media [65]. *Escherichia coli* DH5α, *Acinetobacter baumannii* (Acb), ATCC 19606 and *Bacillus cereus* (Bce) were grown in Luria Bertani (LB) broth and agar. To enrich EVs of Acb and Bce, 1.0 O.D. (at A_600nm_) cultures (in LB, 37 °C, 220 rpm) were washed twice with equal volumes of fresh LB media at ~3200× *g* for 15 min at RT, then sub-cultured to 0.05 O.D. and grown until the required volume of culture reached mid-exponential phase. Typically, 2 L cultures were grown to enrich EVs of Mtb, Msm, Acb and Bce.

### 4.3. EVs Enrichment and Density Gradient Fractionation

Previously described protocols [24,42] were followed with minor modifications. Briefly, mid-exponential phase cultures of Msm and Mtb (~2 L) in Sauton’s media were pelleted down for 20 min at 4 °C at ~8000× *g* (Thermo Fisher Scientific Sorvall RC6 Plus, Waltham, MA, USA). Acb and Bce grown to mid-exponential phase in LB broth were also pelleted down for 20 min at 4 °C at ~8000× *g*. The spent media obtained from each of these in vitro grown cultures were filtered twice (first with 0.45 micron and then by 0.22-micron filters (Merck Millipore, Burlington, MA, USA)). The culture filtrates thus obtained were concentrated through 30-kDa centricon concentrators at 4 °C for 20 min at 4000× *g* (Merck Millipore) to approx. 38 mL each. The concentrates thus achieved were subjected to a two-step centrifugation (SS-34 rotor; Thermo Fisher Scientific Sorvall RC6 Plus, for 20 min at 4 °C, first at 4000× *g* and then at 15,000× *g* to remove any remaining debris. The supernatants were then ultracentrifuged (in 38 mL ultracentrifuge tubes) in cold (4 °C) at 100,000× *g* for 4 h using SW-28 rotor in Optima L-100K Ultracentrifuge (Beckman Coulter, Brea, CA, USA). The pellets thus obtained were first resuspended in 600 µL of HEPES buffer solution (50 mM HEPES and 150 mM NaCl, pH 7.4; pellet left O/N in cold (4 °C)) and then gently pipetted up and down until suspended) and then layered at the bottom of a discontinuous Optiprep (Merck, Burlington, MA, USA) gradient (*w*/*v*; 60—6%; 60%—3.4 mL, 40%, 30%, 20%, and 10%—1 mL each, and 6%—4 mL)) and subjected again to ultracentrifugation (in 14 mL ultracentrifuge tubes) on a swinging bucket SW-40 Ti rotor (Beckman Coulter, USA) in cold (4 °C) at 141,000× *g* for 16 h. One mL fractions that contain EVs (4th to 6th for Msm; 5th to 7th for Mtb; 3rd to 7th for Acb and Bce; confirmed by Transmission Electron Microscope) were collected, combined and diluted in HEPES buffer to a total of 38 mL and ultracentrifuged at 100,000× *g* for 16 h using SW-28 rotor to remove Optiprep from enriched EVs. The enriched EVs pellet was resuspended in 600 µL of HEPES buffer, sonicated for 10 min in a water bath sonicator, particle size and concentration of the samples determined by Nanoparticle Tracking Analysis (NTA) and then frozen as aliquots in −80 °C until used for all downstream experiments. The quantity of total protein in EVs obtained from 2 L cultures was quantified using the Pierce BCA Protein Assay Kit. Approx. 120–150 µg (for Mtb strains—H37Rv and H37Ra) and 80–100 µg (for Msm) protein equivalent of EVs were routinely enriched from 2 L of cultures. Around 150–200 µg protein equivalent of EVs were obtained with 2 L of Acb and Bce cultures. BCA protein estimation kit was routinely used for protein estimation. The manufacturer’s protocol for protein estimation was carefully followed with suitable controls and blanks.

### 4.4. Negative Staining and Transmission Electron Microscopy (TEM) of mEVs

Carbon-coated TEM copper grids (300 mesh; β-Tech, INDIA) were first floated for 10 min on 20 µL droplets of enriched mEVs that were dropped on a clean (wiped prior with 70% ethanol and air-dried) parafilm strip (Thermo Fisher Scientific). The loaded grids were subsequently washed for 30 s by placing them on 20 µL droplets of double-autoclaved water (filtered through 0.22 µm filter). Excess water was blotted out with clean Whatman filter paper (Merck, USA). For negative staining, the grids with Msm-derived EVs were floated for 30 s onto 15 µL droplets of freshly prepared 2% Phospho-tungstic acid (PTA (Sigma Aldrich, USA)—prepared in double-autoclaved filtered (in 0.22 µm filter (MDI, INDIA) water)). Excess negative stain was blotted out with Whatmann filter paper to remove any traces of excess PTA and air-dried before viewing under a Transmission electron microscope (Tecnai 12 BioTWIN, FEI, Hillsboro, OR, USA). Electron micrographs were digitally recorded using a Megaview II (SIS, Germany) digital camera. Image analysis and diameter measurements were performed using an Analysis II (Megaview SIS, Germany) software package.

### 4.5. Nanoparticle Tracking Analysis of mEVs

Particle size and concentration of the samples were determined by NTA using NanoSight NS 500 (Malvern, Worcestershire, UK) and as per the manufacturer’s recommendations. The instrument was equipped with NTA 3.1 analytical software, a high sensitivity sCMOS camera and a 488 nm (blue) laser. All vesicle suspensions were sonicated for 10 min in a water bath sonicator (Branson 5510; Emerson, Brookfield, CT, USA) and subsequently diluted 100-fold in HEPES buffer. Thirty-sec videos of every sample (three min total) were recorded with camera level at 7 and technical triplicates averaged. Software settings for analysis were kept constant for every measurement (screen gain 1, detection threshold 4). HEPES buffer was read as a control before each experiment to ensure that it was free of particles. The laser chamber was always rinsed thrice between every sample reading with particle-free distilled and autoclaved water (Merck, USA).

### 4.6. Systematic Evolution of Ligands through Exponential Enrichment (SELEX)

High-affinity single-stranded DNA (ssDNA) aptamers against Msm EVs were identified using nitrocellulose membrane (NCM)-based SELEX strategy as described by our group [34] with slight modifications. Briefly, the aptamer library was first incubated with a clean NCM for 1 h to remove membrane-binding species from the total population (negative selection). Those ssDNA molecules that did not bind NCM were collected and incubated for 1 h with Msm-derived EVs immobilized (drop-casted) to NCM (positive selection). The unbound and/or loosely bound aptamer species were washed away with a selection buffer (Tris-HCl—10 mM pH 7.5 supplemented with 10 mM MgCl_2_, 25 mM NaCl, 50 mM KCl and 0.1% Tween-20) The tightly bound aptamer species were then eluted by heating the NCM on a dry bath at 92 °C. The eluted pool of aptamers was then amplified by PCR with primers [34]; ssDNA was prepared from this enriched pool using alkaline lysis method coupled with Urea-PAGE as described earlier [34].

These ssDNA molecules were then used as a screening library for the next round of SELEX. A total of six reiterations of SELEX were performed to shortlist the high-affinity binders. In every round, the stringency of selection was enhanced by increasing the incubation time for negative selection and decreasing the incubation time for positive selection. The stringency of washing was also enhanced by increasing the strength of Tween-20 (from 0.25% to 1%) in the SB wash buffer. Additionally, we also reduced the amount of mEVs (from 20 to 2 µg) in successive rounds of SELEX. After six rounds of SELEX, the archived aptamer pool from rounds 1, 2, 4 and 6 was assessed for its ability to bind MVs in an aptamer linked immobilized sorbent assay (ALISA, a modified version of ELISA). Finally, the aptamer pool obtained after round 6 of SELEX was cloned in a TA cloning vector (pTZ57R/T, Thermofisher Scientific) and transformed into *E. coli* DH5α competent cells [5,7]. The aptamer clones were picked and sent for sequencing. DNA sequencing data were analysed by CLUSTAL W and Bioedit sequence alignment editor [66] to study the primary sequence homology among obtained aptamer candidates.

### 4.7. Aptamer Linked Immobilized Sorbent Assay (ALISA)

The binding propensity of each monoclonal aptamer candidate from the sequenced aptamer pool/were assessed by employing ALISA [35,67]. Briefly, 500 ng Msm EVs were coated in a MaxiSorp™ 96 well plate using 100 mm carbonate–bicarbonate buffer, pH 9.6 for 2 h at 37 °C. The plate was then blocked either with 5.0% bovine serum albumin (BSA) or skim milk for 60 min at RT. The plate was then washed with SB and 100 pmole of 5′ biotin-labelled aptamer in question was added and the plate was incubated at RT for 1 h. The excess unbound aptamer molecules were washed away with wash buffer (SB supplemented with 0.25% Tween-20 *v*/*v*). The Msm EVs-bound aptamers were then probed with 1:3000 streptavidin-conjugated horseradish peroxidase (HRP) and aptamer binding was visualized with 100 µL of OptEIA TMB substrate, (3,3′,5,5′-tetramethylbenzidine; BD Biosciences, USA). After 5 min incubation, the reaction was stopped using 5% H_2_SO_4_ and O.D. was measured at 450 nm. The only SB buffer-coated wells served as control. The data was plotted as a difference in O.D. (ΔO.D.; the actual O.D. value of Msm EVs coated well minus O.D. value of only buffer-coated well) at 450 nm.

### 4.8. Assessment of Cross-Reactivity of Aptamers

To assess the cross-reactivity of shortlisted aptamer candidates, ALISA (as described in previous section) was employed. Briefly, 500 ng/well of EVs derived from Msm, *Acinetobacter baumannii* (Acb, Gram-negative pathogen) and *Bacillus cereus* (Bce, Gram-positive pathogen) were coated onto 96 well plates and ALISA was performed. Similarly, to assess if the Msm EVs-specific aptamers could also recognize Mtb-derived (both pathogenic, H37Rv and attenuated, H37Ra) EVs, 500 ng/well of EVs from Msm and Mtb were coated onto 96 well plates and ALISA performed.

### 4.9. Evaluation of Aptamers Affinity to Proteinase K-Treated Msm EVs

To evaluate if the shortlisted high-affinity binders primarily bound to surface proteins of Msm EVs, 500 ng of Msm-derived EVs were coated onto the 96 well plates and then treated with 300 ng of Proteinase K for 30 min. Then, excess Proteinase K was washed off with SB. Affinity of the shortlisted aptamers to untreated and Proteinase K-treated Msm EVs specific was assessed with ALISA, as described earlier.

### 4.10. Binding Affinity of Aptamers

The binding affinity of the best performing Msm EVs-specific ssDNA aptamers (apt-3 and -21) were determined by identifying their dissociation constants (Kd). Towards that, 500 ng/well of Msm EVs were coated onto 96 well plates as described before. After blocking the unbound surface of wells with 5% BSA (1 h, RT; excess washed off), different concentrations (2 nM to 500 nM) of 5′ biotin-labelled apt-3 and apt-21 aptamers were taken, and the rest of the ALISA procedure was the same as described in Section 4.7. The aptamer binding (ΔOD 450 nm values) was plotted as a function of aptamer concentration (Graph Pad Prism^®^ software, v5.0) and dissociation constant (Kd) was estimated using non-linear regression equation Y = (B max * X)/(Kd + X), where Y is ΔOD 450 nm, B max is maximum binding, X is the concentration of aptamer, Kd is the dissociation constant.

### 4.11. In Vitro Cell Culture Experiments

Cell culture work with human monocyte cell line THP-1 (from Cell repository, NCCS, Pune, India; authenticated by STR profiling at Lifecode Technologies Pvt. Ltd., INDIA; also verified by multiplex-PCR for no cross-contamination with cell lines derived from Chinese hamster, grivet monkey, rat and mouse) was performed as in Nishant Sharma et al., 2019 [65]. Briefly, as undifferentiated monocytes, THP-1 were maintained in sterile RPMI 1640 medium supplemented with 10% fetal bovine serum (FBS) and 1% penicillin–streptomycin at 37 °C, 5% CO_2_. They were differentiated into macrophages by supplementing with 20 nM phorbol-12-miristate-13-acetate (PMA). After 72 h, differentiated cells were washed thrice with sterile, 1× PBS (at 37 °C), pH 7.4 and then used for downstream studies.

### 4.12. Confocal Microscopy

Undifferentiated THP-1 seeded to 10^5^/well in two-chamber slides were differentiated with 20 nM PMA for 72 h. Cells were rinsed thrice with pre-warmed 1× PBS, pH 7.4 to remove any traces of PMA. To assess non-specific binding of apt-3 and -21 (labelled with Cy5) to THP-1, either 100 or 300 picomoles were added directly to differentiated THP-1. After washing the excess, cells were fixed using BD cytofix kit as per the manufacturer’s instructions. Cells were then rinsed thrice with pre-warmed 1× PBS, pH 7.4 and mounted with DAPI to observe under confocal microscope, FV 3000 (Olympus, Shinjuku, Tokyo, Japan).

After shortlisting the best performing aptamer, to visualize mEVs in macrophages, we exposed the differentiated THP-1 at 1:1000 ratio with mEVs (derived from MtbRv, MtbRa and Msm) and incubated for 2 h at 37 °C and 5% CO_2_. The spent media was aspirated and replaced with fresh pre-warmed RPMI containing 100 picomoles of apt-21 labelled with Cy5. After 2 h, the spent media was again aspirated, and THP-1 containing mEVs and apt-21 were rinsed thrice with pre-warmed 1× PBS, pH 7.4 to remove excess unbound aptamer. Cells were fixed using BD cytofix/cyto permeabilization kit as per the manufacturer’s instructions. The cells were rinsed thrice with pre-warmed 1× PBS, pH 7.4 and mounted with DAPI to observe under confocal microscope, FV 3000).

For infecting THP-1 with GFP-expressing H37Ra, in vitro grown bacteria (~0.3 O.D. (A600 nm)) were pelleted down at 4000 rpm, RT and for 10 min. They were then washed with RPMI (having 2 mM L-Glutamine, 10 mM HEPES and 10% FBS) and filtered through a 5 μm syringe filter (Merck, USA) to obtain single-cell suspension. THP-1 were infected at MOI = 1:10 for 4 h at 37 °C, and 5% CO_2_. The uninfected controls and infected cells were washed thrice with pre-warmed 1× PBS, pH 7.4 and incubated for 2 h in fresh, pre-warmed RPMI containing Amikacin (200 μg/mL) to eliminate any extracellular bacteria. The cells were again washed thrice with pre-warmed 1× PBS, pH 7.4 and incubated for 2 h with pre-warmed RPMI containing 100 pmoles of apt-21. The spent media was then aspirated, and cells were rinsed thrice with pre-warmed 1× PBS, pH 7.4 to remove all unbound aptamer. Cells were fixed using a BD cytofix/cytopermeabilisation kit as per the manufacturer’s instructions. The cells were then rinsed thrice with pre-warmed 1× PBS, pH 7.4 and mounted with DAPI to observe under confocal microscope, FV 3000.

### 4.13. Statistical Analysis

Statistical analysis was performed using Graph Pad Prism v9.0 software. All experimental data are represented as mean ± SD. Where necessary, the statistical significance was determined by using either the unpaired two-tailed Student’s *t*-test or ANOVA. *p* values < 0.05 were considered statistically significant and represented as **** for *p* < 0.0001 (for Student’s *t*-test) or *** *p* < 0.0006 and **** *p* < 0.0001 (for ANOVA).

## 5. Conclusions

Employing a SELEX-based reiterated screening on mycobacterial membrane-derived extracellular vesicles (mEVs),we identified a best performing, high affinity-binding aptamer viz. apt-21, that binds mEVs with low nanomolar affinity and exhibits no cross reactivity to membrane-derived vesicles of other Gram -ve (Acb) and Gram +ve (Bce) bacterial pathogens and to THP-1 macrophages. Our analyses led us to recognize that the primary aptatopes are mEVs-specific surface bound proteins. This tool, thus aided us to not only clearly visualize mEVs that mycobacteria generate in axenic cultures, but also clearly visualize mEVs upon their internalization by THP-1 macrophages or upon their release directly into THP-1 macrophages by infecting mycobacteria. Together, our work for the first time demonstrates release of mEVs in vitro post infection of macrophages by mycobacteria. We anticipate that in the near future, this tool holds great potency in delineating mEVs-derived pathogenic functions and in acting as a non-invasive clinical diagnostic tool.

## Data Availability

Data is contained within the article.

## Supplementary Materials

The following are available online at https://www.mdpi.com/article/10.3390/ph15010045/s1, Figure S1: SELEX-based enrichment rapidly aids shortlisting of mEVs-binding aptamers, Figure S2: Characterisation of M. smegmatis-derived EVs using Transmission Electron Microscopy (TEM), Figure S3: Aptamer 21 detects EVs released by Mtb post infection in THP-1.
