# Supplementary material for: Development of DNA Aptamers to Visualize Release of Mycobacterial Membrane-Derived Extracellular Vesicles in Infected Macrophages
Source: Pharmaceuticals (Basel). 2021 Dec 29;15(1):45. doi: 10.3390/ph15010045 (PMC8779091; doi:10.3390/ph15010045)
Supplement: Supplementary file 1 [file pharmaceuticals-15-00045-s001.zip › pharmaceuticals-1457671-supplementary.pdf]

Supplementary Materials:

Materials and methods:

Negative Staining and Transmission Electron Microscopy (TEM) of mEVs.

Carbon-coated TEM copper grids (300 mesh; β-Tech, INDIA) were first floated for 10 min on 20 μl droplets of enriched mEVs that were dropped on a clean (wiped prior with 70% ethanol and air dried) parafilm strip (Thermo Fisher Scientific). The sample loaded grids were subsequently washed for 30 s by placing them on 20 μl droplets of double-autoclaved water (also filtered through 0.22 μm filter). Excess water was blotted out with clean Whatmann filter paper (Merck, USA). For negative staining, the grids with Msm-derived EVs were floated for 30 s onto 15 μl droplets of freshly prepared 2% Phospho-tungstic acid (PTA (Sigma Aldrich, USA) – prepared in double-autoclaved filtered (in 0.22 μm filter (MDI, INDIA) water). Excess negative stain was blotted out with Whatmann filter paper and air dried before viewing under a Transmission electron microscope (Tecnai 12 BioTWIN, FEI, Netherlands). Electron micrographs were digitally recorded using a Megaview II (SIS, Germany) digital camera. Image analysis and diameter measurements were performed using an Analysis II (Megaview SIS, Germany) software package.

Nanoparticle Tracking Analysis of mEVs.

Particle size and concentration of the samples were determined by Nanoparticle Tracking Analysis (NTA) using NanoSight NS 500 (Malvern, UK; as per manufacturer's recommendations). The instrument was equipped with NTA 3.1 analytical software, a high sensitivity sCMOS camera and a 488 nm (blue) laser. All vesicle suspensions were sonicated for 10 min in a water bath sonicator (Branson 5510; Emerson, USA) and subsequently diluted 100-fold in HEPES buffer (50 mM HEPES and 150 mM NaCl, pH 7.4). Thirty sec videos of every sample (3 min total) were recorded with camera settings at level '7' and technical triplicates measurements averaged. Software settings for analysis were kept constant for every measurement (screen gain setting at '1', detection threshold setting at '4'). HEPES buffer alone was read as control before recording each sample reading (particles 'free' control). The laser chamber was rinsed atleast thrice between every sample reading with particle-free distilled, double-autoclaved and filtered water (Merck, USA).

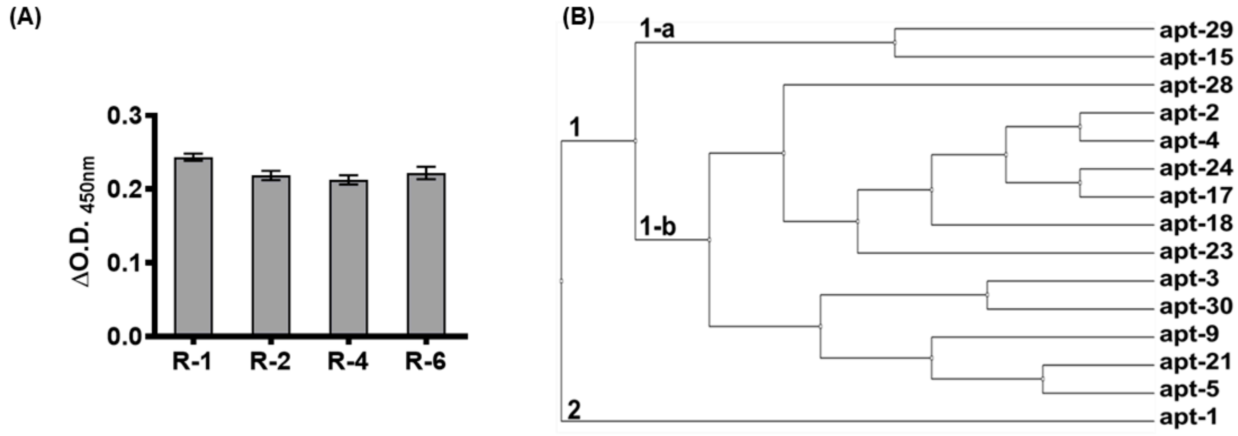

Figure S1. ***SELEX-based enrichment rapidly aids shortlisting of mEVs-binding aptamers*****. (A):** Enrichment of mEVs-binding aptamers with respect to SELEX rounds (R-1 to R-6; **X-axis**); bars represent mean ± SD; The propensity of aptamers binding was assessed by employing ALISA (***Aptamer Linked Immobilized Sorbent Assay***; see main Materials and Methods for detailed protocol). The Msm EVs-bound aptamers were probed with 1:3000 streptavidin-conjugated Horse Radish Peroxidase (HRP) and aptamer binding visualized with 100 µL of OptEIA TMB substrate, (3,3′ ,5,5′ -tetramethylbenzidine; BD Biosciences, USA). After five min incubation, the reaction was stopped using 5 % H_2SO_4 and optical density (O.D.) was measured at 450 nm. The only buffer (100 mM Carbonate-Bicarbonate buffer, pH 9.6) coated wells served as control. The data was plotted as a difference in O.D. (ΔO.D.; the actual O.D. value of Msm EVs coated well minus O.D. value of only buffer coated well; **Y-axis**) at 450 nm. **(B):** Phylogenetic tree (generated using CLUSTAL W tool) shows clustering of sequenced mEVs-binding aptamer candidates (numbered in black – to the right) based on their primary sequence homology. SELEX-derived aptamers are clustered together in two preponderant groups **(1 & 2)** with group 1 being prominent **(1-a & 1-b)**.

(A)

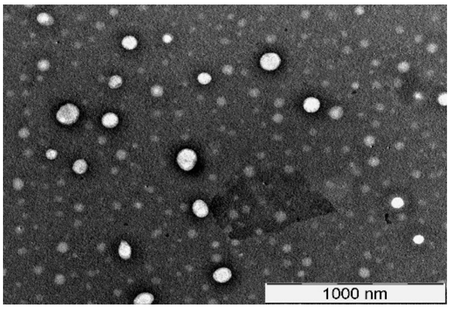

(B)

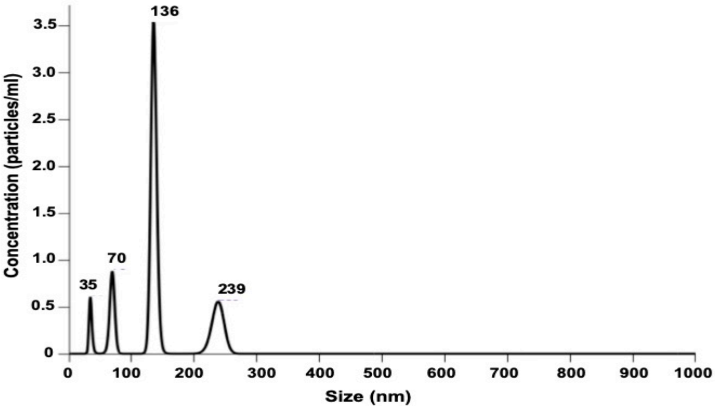

Figure S2. ***Characterisation of M. smegmatis-derived EVs*** using Transmission Electron Microscopy (TEM) **(A)** and Nanoparticle Tracking Analysis (NTA) **(B)**. **(A):** Representative TEM image showing intact and heterogenous population of mEVs enriched from *M. smegmatis* (Msm), and negatively stained with 2% Phosphotungstic acid. Approx. 20 µl of the enriched EVs (enriched from filtered and concentrated spent media of mid-log cultures of Msm (~ 2.5 O.D. (at A_{600nm})) that were ultracentrifuged and separated by Optiprep density gradient ultracentrifugation – detailed protocol in main Materials and Methods) were used for loading onto carbon grids. The images were acquired, analysed and processed using Analysis II (Megaview SIS, Germany) software. **(B)**: Representative NTA graph showing size in nm (approx. diameter of the spherical structures; **X-axis**) vs concentration (particles/ml; **Y-axis**) of enriched EVs. Approx. 20 µl aliquot of enriched EVs was made up to 1 ml with HEPES buffer and injected (using sterilised 1 ml syringe) into the NTA sample chamber. Concentrations of EVs in the sample was analysed thrice (technical triplicates) using NanoSight Tracking Analysis NS 500 (30 sec videos recorded with camera level at 7 and averaged). The NTA images were analysed by the software NanoSight NTA 3.1.

(i)

(ii)

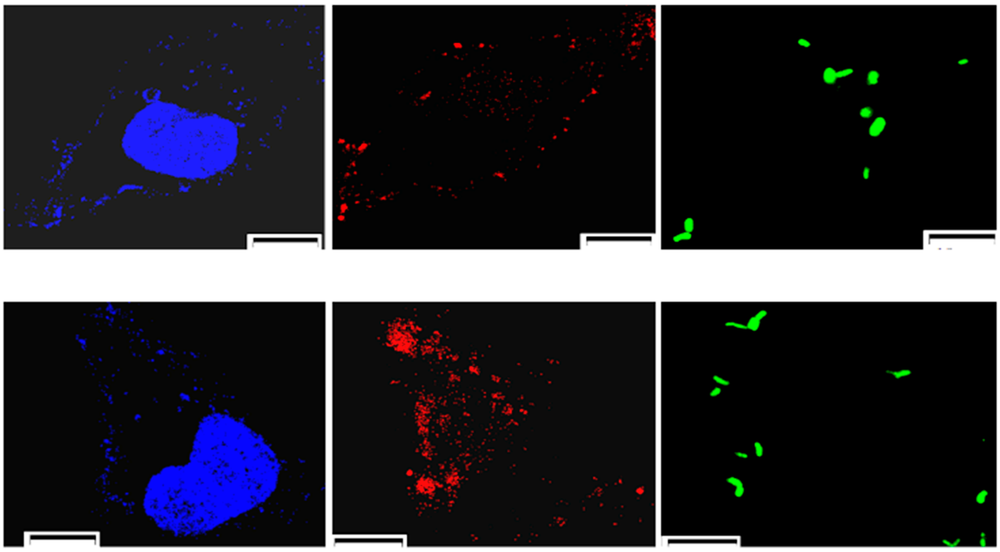

Figure S3. ***Aptamer 21 detects EVs released by Mtb post infection in THP-1.*** PMA-differentiated THP-1 were infected with GFP-expressing Mtb (H37Ra) for 4 h **(i & ii)**. Extracellular Mtb were killed with Amikacin (200 ug/ml) and then, the infected THP-1 incubated for 2 h with Cy5-labelled apt-21 (100 pmoles). Excess aptamer was removed with three washes of pre-warmed 1X PBS (pH 7.4). Cells were fixed with BD cytofix/cyto-permeabilization kit and mounted with Prolong Gold antifade DAPI to observe under the confocal microscope. The single channel images were captured at 60X magnification and zoomed to 120X. Blue, DAPI (nuclear staining); Green, Mtb (H37Ra); Red, apt-21. Black bars in (i) and (ii) indicate scale bar of 10 µm.
